# Current Concepts of Laser–Oral Tissue Interaction

**DOI:** 10.3390/dj8030061

**Published:** 2020-06-28

**Authors:** Steven Parker, Mark Cronshaw, Eugenia Anagnostaki, Valina Mylona, Edward Lynch, Martin Grootveld

**Affiliations:** 1 Leicester School of Pharmacy, De Montfort University, Leicester LE1 9BH, UK; Drmarkcronshaw@outlook.com (M.C.); eugenia.anagnostakis@my365.dmu.ac.uk (E.A.); vasiliki.mylona@my365.dmu.ac.uk (V.M.); edward.lynch@hotmail.com (E.L.); mgrootveld@dmu.ac.uk (M.G.); 2 School of Dental Medicine, University of Nevada Las Vegas, Las Vegas, NV 89106, USA

**Keywords:** dentistry, laser, laser–tissue interaction, optical properties of tissues, photobiomodulation, photothermolysis

## Abstract

Fundamental to the adjunctive use of laser photonic energy for delivering therapy and tissue management, is the ability of the incident energy to be absorbed by target tissues. The aim of this review is to examine the differential performance of the separate components of oral hard and soft tissues when exposed to laser photonic irradiance of variable wavelengths and power values. Through an examination of peer-reviewed published data and materials, the interaction of laser photonic energy and target tissues are explored in detail. Varying laser wavelength emissions relative to anatomical structures explores the ability to optimise laser–tissue interactions, and also identifies possible risk scenarios as they apply to adjacent non-target structures. The concepts and practical aspects of laser photonic energy interactions with target oral tissues are clearly demonstrated. Emphasis was placed on optimising the minimum level of laser power delivery in order to achieve a desired tissue effect, whilst minimising the risk or outcome of collateral tissue damage.

## 1. Introduction

The oral cavity is a complex environment, both structurally and functionally, where hard and soft tissues exist in close proximity. Additionally, all oral tissue surfaces are subject to contact with bacteria- and bacterial catabolite-laden saliva. Oral tissues can be subjected to laser treatment exposure, but the biophysics governing laser–tissue interactions demands a knowledge of all factors involved in the delivery of this modality.

Laser photonic energy has the unique properties of spatial and temporal coherence of wave propagation, together with the emission monochromaticity derived through a single wavelength value. In consequence, with appropriate delivery parameters, it is possible to deliver selective laser–tissue interactions, and to maximise the light dose effectiveness [1].

**Figure 1:**
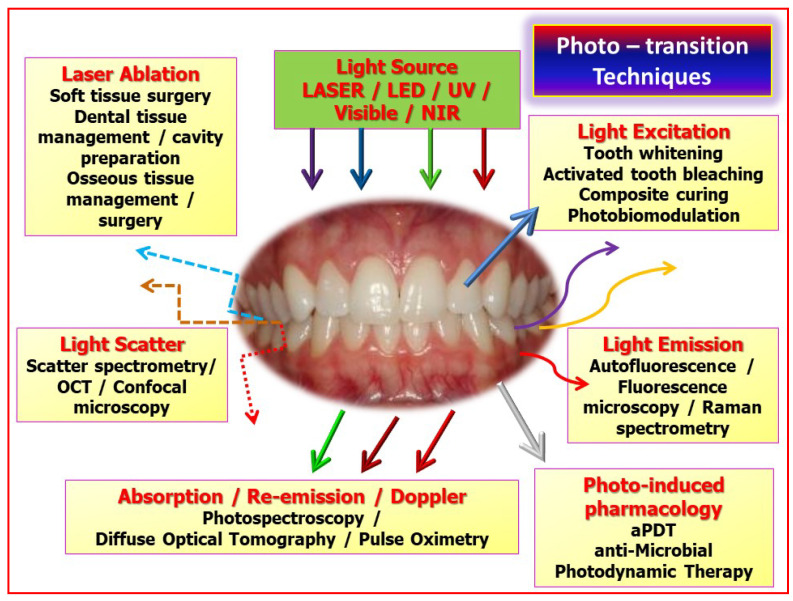
An overview of the manipulation of incident photonic energy, such as laser light as an adjunct to screening, diagnostic and therapeutic clinical activity. Key: LED—light emitting diode, UV—ultra-violet, NIR—near infra-red, with multi-wavelength representation as vertical incident arrows. OCT—optical coherence tomography, aPDT—antimicrobial photodynamic therapy. Re-emission, as direct beam or scattered irradiance is represented by complete and dotted arrows, either straight or non-linear.

Depending on the level of incident energy, the consequent transfer to another form can occur, such as thermal energy, fluorescence, sound emission and the promotion of chemical pathways within tissue cellular environments. These interactions are summarised in Figure 1.

**Figure 2:**
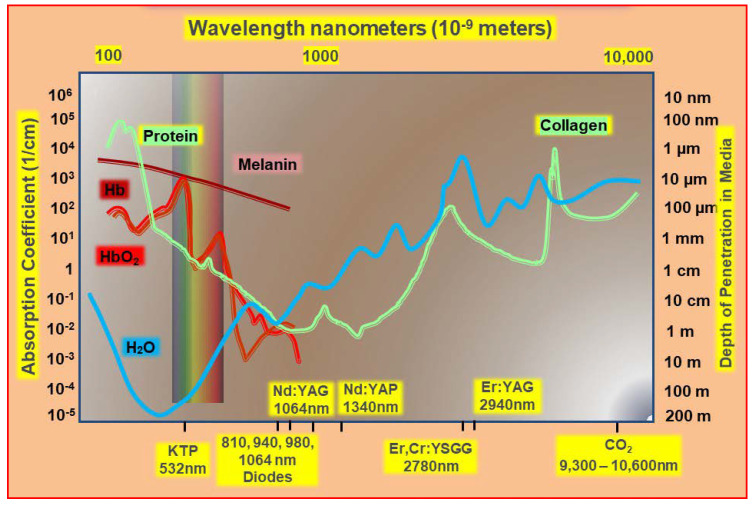
Absorption spectral profiles of major dental/oral structural elements and chromophores associated with soft tissue management. Absorbance is shown relative to wavelength of irradiation. The depth of penetration is shown as 1/absorbance. Original data and graphics: Parker S. Data source: Parker, S; et al. Laser Essentials for the Dental Practitioner: Foundation Knowledge—Construction, Modes of Operation and Safety. EC Dental Science **2019,** 18.9, 2020–2027 [2].

**Figure 3:**
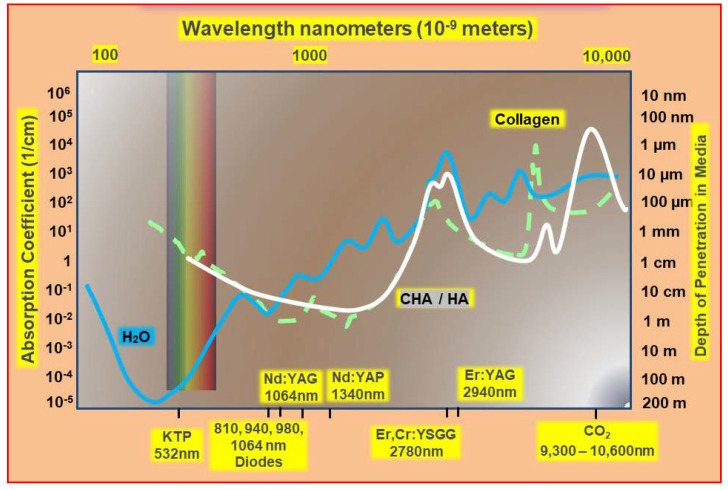
Absorption spectral profiles curves of major tissue elements associated with bone and dental hard tissue management. Absorbance is shown relative to wavelength of irradiation. Key: water (blue), protein/collagen (green), hydroxyapatite—HA and carbonated HA (white). Original data and graphics: Parker S. Data source: Parker, S; et al. Laser Essentials for the Dental Practitioner: Foundation Knowledge—Construction, Modes of Operation and Safety. EC Dental Science **2019,** 18.9, 2020–2027 [2].

The ability to predict the degree of laser–tissue interaction experienced may pose some difficulties for the clinician, notably through limited target access, choice of laser wavelength, and the amount of power required to achieve desired changes in the target tissue. As the prime factor governing interaction, the absorption coefficient of any tissue element is a function of the degree of energy attenuation of a chosen incident laser wavelength; within the wide range of laser wavelengths available in dentistry, absorption coefficient curves as demonstrated in Figure 2 and Figure 3 are an expression of the relative performance of each tissular element across the spectrum of incident photonic irradiation.

Within tissue elements, there may be molecules or molecular species termed chromophores. A chromophore is defined as “a chemical group (molecule or molecular species) that absorbs light, or at least shows some absorbance in the visible region of the electromagnetic spectrum and so imparts colour to the molecule”. 

Each chromophore has molecular structure and the structure, atomic configuration and inter-atomic binding energy at body temperature may define a “ground state” [3]. In achieving ablative laser surgery in soft or hard tissue clinical dentistry, the effect of absorption of incident energy is the conversion to thermal energy and consequent tissue ablation through thermal rise—a process known as photothermolysis. Assuming that absorption can occur, the relationship between the level of incident photonic energy, the density of such energy within an irradiated area, and the exposure time (instantaneous and/or over a sustained or interrupted period of time), may enable the clinician to influence the type of laser–tissue interaction. By reducing the exposure time and consequent interaction to micro-seconds and on to femto-seconds, successively higher peak power densities above 10^6−10^ W/cm^2^ can be obtained. At such powerful levels, the intensity of energy is so great that electromagnetic fields (plasma) develop around the interaction and are sufficient to markedly degrade target molecules, a process known as photoplasmolysis [4].

**Figure 4:**
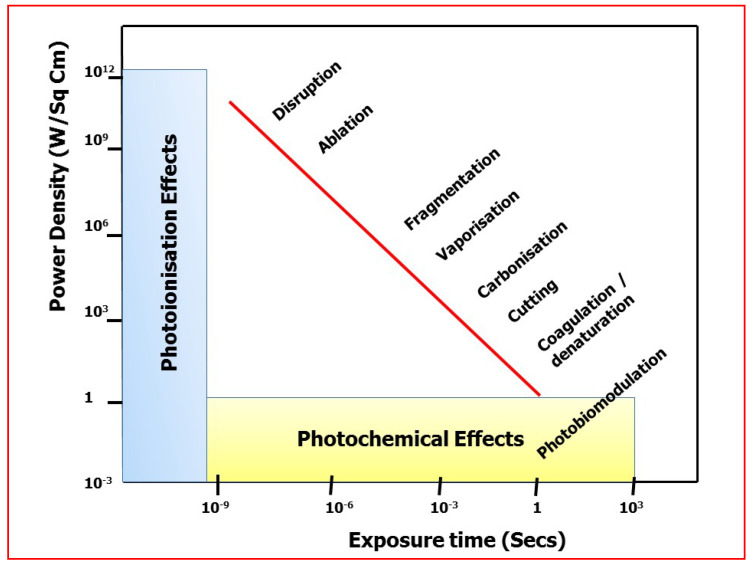
Relationship between incident photonic power density and exposure time (seconds). Changes in the two components of laser–tissue interactions—power density (irradiance) and exposure time, may affect the interaction level. Original graphics: Parker S. Data source: Boulnois, J-L. Laser Med. Sci. **1986,** (1), 47–66 [5].

Conversely, very low irradiance over extended time periods may give rise to energised biochemical pathways associated with tissue health and reparative capabilities, and this represents the core of understanding of photobiomodulation (PBM). Figure 4 provides a graphic representation of the relationship between power density and exposure time [5].

## 2. Laser Photonic Energy

**Figure 5:**
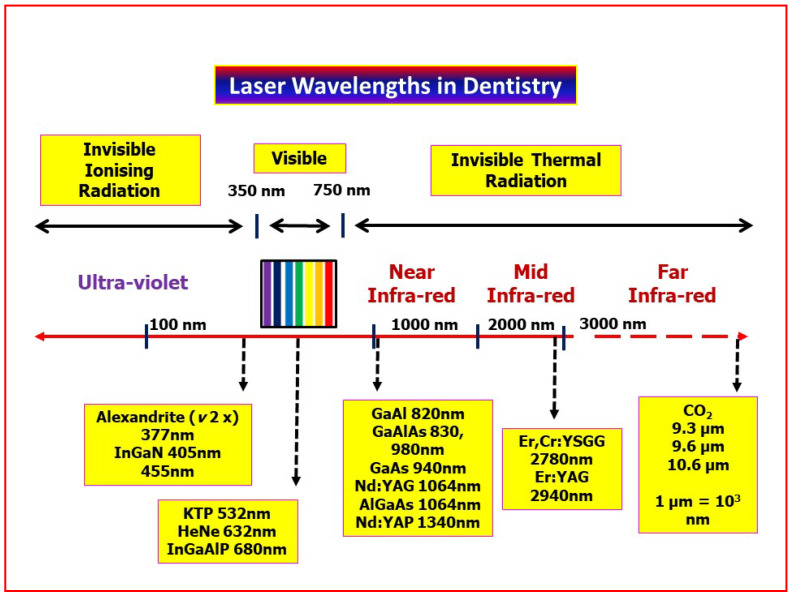
Laser wavelengths in common use in clinical dentistry, arranged according to wavelength in nanometers (10^−9^ m), from blue-visible to the far infra-red regions.

Laser photonic energy is derived through the emission of photons from an energised source. The photon (electromagnetic) energy is derived from the parent stimulated atomic source within the active medium, and its propagation within a stream of photons provides an identical emission waveform (coherence); each photon within the stream has an identical energy value (monochromatic or single wavelength). The relationship between common laser wavelengths within the electromagnetic spectrum is represented in Figure 5.

The energy of emitted photons is expressed in Joules, or a derived equivalence, eV (a unit of energy equal to the work done on an electron in accelerating it through a potential difference of one volt), where 1 eV = 1.602 × 10^−19^ J. Since photonic energy and wavelength (λ) have an inverse proportional relationship, photons emitted from different active medium sources will have differing energy values. A basic expression of this relationship is demonstrated in equation 1, in which *h* = Plank’s constant, c = speed of light and E = photon energy in eV.

λ = *h*c/E (1)

It is possible to evaluate energy-equivalent values for the laser wavelengths commonly used in dentistry, for example, photons of wavelength 1240 nm (near-infra-red region) equate to an eV value of 1.0, whereas an eV value of 2.0 is represented by a wavelength of 621 nm (visible-red region); 0.13 eV corresponds to 9600 nm [6]. 

Table 1 provides an overview of laser wavelengths commonly used in dentistry with corresponding photonic values:

A simplistic view of one of the many graphic representations of the inter-relationships between target tissue elements, incident laser wavelengths and relative absorption potential, would suggest that laser photonic energy is capable of ablative interaction with target tissue elements [7]. If external energy is applied, vibrational and spin phenomena are induced—initially reversible to the ground state, but with increased applied energy, a point may be reached where molecular disruption is sufficient to overcome the forces binding atoms or macromolecules together [8]. Incident photonic energy may induce a thermal rise (energy conversion), and examples of the effects of ascending energy levels above an “ablation” threshold include protein dissociation (*ca.* 60 °C) and water vaporisation. True photonic ablation of a target molecule therefore represents incident energy sufficiently intense to break interatomic binding forces, and this is termed the dissociation energy. Table 2. Provides examples of commonly found tissue element molecules and the dissociation energy value required to break the interatomic bond.

When comparing data in Table 1 and Table 2*,* almost none of the popular laser photonic energies in dentistry are capable of direct intra-molecular bond cleavage, and one may conclude that dental lasers cannot ablate target oral tissues through the use of empirical photonic energies. Such a belief is magnified when the binding (ionic) lattice energies of crystalline carbonated hydroxyapatite are exposed to the mid-IR laser wavelengths (Er,Cr:YSGG, Er:YAG); indeed, the photonic energy values may be considered very small when compared to that required for the dissociation of hard dental tissue [9].

Although individual photons possess insufficient energy to cleave target molecules, even the briefest of laser emissions will deliver millions of individual photons, and with each successive photon absorbed, the gradual build-up of transferred energy causes increasing target molecular vibrations, up to a point where sufficiently high power density (i.e., energy density within an ultra-short time duration) and consequent thermal rises, drives molecular fragmentation and structural ablation. 

Molecular disruption is not the objective when considering the many sub-ablative applications of laser photonic energy within PBM, diagnostic and para-chemical antimicrobial applications. With this group of applications, the modulation of target structural molecules or biochemical pathways remains the objective within a general enhancement of cellular and intercellular activities, with consequent benefits being derived through the healing, analgesic and anti-inflammatory applications of sub-ablative laser irradiation.

When laser photonic energy is delivered and interacts with a tissue medium, three possible pathways exist to account for what arises from the delivered light energy:

(1) The commonest pathway when light is absorbed by living tissue is internal conversion from incident photonic to thermal energy. In surgical laser use, the resulting thermal rise is near-instantaneous and considerable, and rapidly leads to conductive thermal energy into surrounding tissue. With oral soft tissues and visible/near-IR laser wavelengths, the absorption by tissue chromophores gives rise to protein denaturation and secondary vaporisation of interstitial water. Through this, a visible ablation and vaporisation of target tissue occurs [10].

With longer mid- and far-IR laser wavelengths, the prime molecule affected by irradiation in both soft and hard oral tissues is highly abundant water. Ablation of tissue is achieved through the near-instantaneous vaporisation of interstitial water, leading to ‘explosive’ fragmentation of tissue structure. With hard osseous/dental tissue, this interaction can be quite dramatic [10].

(2) Laser photonic energy values below target tissue ablation levels may result in fluorescence. Fluorescence is a luminescence (re-emission) of light in which the absorption of a photon by a molecule triggers the emission of another photon with a longer wavelength from that molecule. This action provides the basis for optical scanning techniques employed for caries detection in enamel and dentine, and also tomographic techniques utilised in the scanning of soft tissues for neoplastic change.

(3) The third pathway is broadly termed photochemistry [11]. In view of the energy of the photons involved, covalent bonds primarily remain unbroken. However, the energy may be sufficient for a first excited singlet state to be formed, and this can then undergo intersystem crossing to the long-lived triplet state of the scavenger. Through energy transfer, adjacent diatomic interstitial tissue oxygen (O_2_) molecules may undergo redox interconversions to form reactive oxygen species (ROS, such as superoxide anion) or singlet oxygen (^1^O_2_). Singlet oxygen is an ultra-short-lived product of the parent molecule that can cause cell apoptosis through oxidative stress. Such actions can be commonly encountered in photodynamic therapies, where an intermediary chemical—photosensitiser—is employed to direct energy transfer to target bacterial or alternative cellular sites [12,13].

Electron transfer reactions are highly important in the host cell mitochondrial membrane, where the consequence of photon irradiation may be increased production of ATP [14]. An additional photochemical pathway that can occur after the absorption of a red or NIR photon within a host cell is the dissociation of a co-ordinated ligand from its metal ion coordination centre as an active site within an enzyme. One well known candidate for this pathway is the complexation of nitric oxide (NO^●^) to the iron-and copper ion-containing redox centres in unit IV of the mitochondrial respiratory chain, otherwise known as cytochrome c oxidase. Such an action may induce an increase in intracellular pH and generate ATP, and has been cited, as has been the release of increments of molecularly-bound NO^●^ and intracellular ROS, as basic photobiomodulation cellular theory for the actions of low-level lasers. 

## 3. Laser–Tissue Interaction: Oral Soft Tissue

Although diverse in structural components, oral soft tissue is a mixture of endothelial collagenous tissue with blood and lymph vessels, muscle, site-specific mucous and salivary gland tissues, nerves and other anatomical structures. All oral soft tissue appending to the lips, buccal cavity and oropharynx is surrounded by epithelial tissue—termed the oral mucous membrane (of varying levels of keratinisation) in order to protect structures against regular exposure to physical wear (gingiva, hard palate and dorsum of the tongue covered with keratinised epithelium), or other sites where abrasion is somewhat lower (lips, oral vestibule, ventral tongue and floor of mouth and soft palate). Laser-sensitive biomolecules include tissue water, proteins, chromophoric molecules and the haem prosthetic group of blood haemoglobin species.

**Figure 6:**
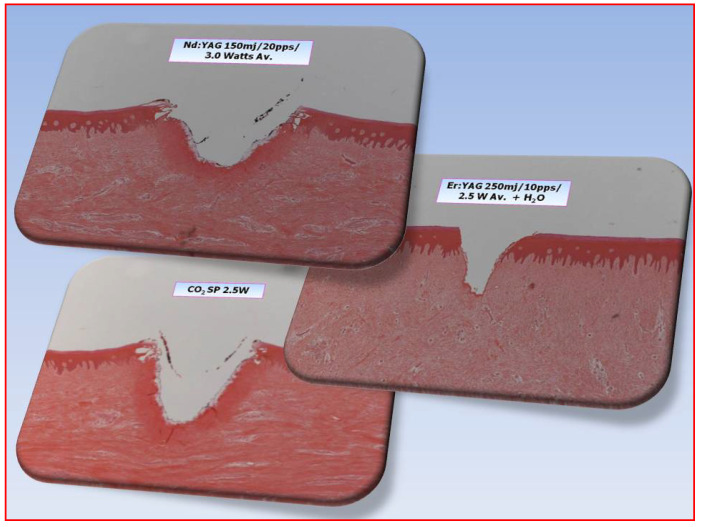
Comparative light micrographs of laser interaction with porcine oral soft tissue. Top: Nd:YAG 1064 nm (an example of the shorter wavelengths employed), causes a wider, crater-shaped area of ablation, with some areas of thermal conduction. Right: Longer wavelengths such as FRP Er:YAG 2940 nm create a sharper “V” shaped incision, whereas Bottom: CO_2_ 10,600 nm, being a gated CW emission, results in some features of the other two—surface configuration ascribable to absorption in water, but some thermal spread arising from a comparative lack of thermal relaxation.

In clinical dentistry, laser applications may involve visible and near-IR wavelengths (absorbed by chromophores, haemoglobin and other proteins), such as the diverse “diode” group (wavelength range 405–1064 nm), mid-infra-red lasers (erbium, chromium: YSGG 2780 nm and erbium: YAG 2940 nm), and the far IR CO_2_ wavelength range of 9300–10,600 nm that can target tissue water. The difference in target laser light scavengers as a function of wavelength will affect both the degree of tissue penetration and essential ablation effect; water, being a constituent of all living matter, will give rise to a surface-mediated ”V-shaped” interaction with longer wavelengths, whereas chromophore and protein interactions that are predominant with shorter wavelengths will result in a wider, crater-shaped area of ablation—Figure 6.

The power delivery (emission mode) of the early dental lasers was either continuous wave or, in the case of Nd:YAG 1064 nm, micro-pulsed. The disadvantage of the continuous wave delivery was the absence of thermal relaxation, which is required to prevent the gradual and damaging build-up of thermal energy into tissue surrounding the target site. Current technology has allowed the development of chopped or “gated” delivery of an inherent continuous wave, allowing millisecond (ms) and some microsecond (µs) bursts of energy. This has helped to refine the overall “average” power delivery. Irrespective of the emission mode, for oral soft tissue surgery to involve gingiva, oral mucous membrane and associated mucous gland tissue, frenal tissue and other benign soft tissue pathologies, there is an adequate opportunity to deliver predictable laser surgery with mean power ranges of 0.8–2.0 W; indeed, the use of considerably higher average power values to ablate or incise tissue may result in collateral tissue damage. A possible exception may be the use of non-contact irradiation of superficial venous haemangiomata, where greater (> 5 W) mean power levels can be employed [15].

The benefits of laser-mediated soft tissue surgery are claimed to be haemostasis and incisional sterility, with the reduced/absent requirement for sutures. Within the applications referenced above, the power values would be sufficient to seal blood and lymphatic vessels of < 0.5 mm diameter [16], and within such limitations, incisional haemostasis offers a near-ubiquitous advantage of visible and near-IR laser use. Additionally, the use of the erbium family and carbon dioxide laser wavelengths, albeit without co-axial water spray, may induce a significant thermal rise within the surgical site sufficient to also seal small vessels and thus avoid dressings or sutures.

Strict sterility of the surgical site is an ideal requirement, but contamination by the normal oral flora may be considered unavoidable. The incisional temperature will exceed 100 °C, and even approach 150–200 °C, and hence would provide a prior level of sterility. An improved representation would be a “significant pathogen reduction”. Of considerable advantage, however, is the formation of a denatured plasma and collagen matrix—a so-called “coagulum” [17], that provides initial sealing of the wound and gradually softens through absorption of saliva throughout a 72–96 hr. period before becoming detached, leaving early endothelial and epithelial cellular ingrowth to initiate healing. Additionally, a feature often observed with laser-mediated soft tissue incisions is the absence of scarring [18], although healing will always proceed via secondary intention in view of tissue volume loss during surgery, and the non-apposition of cut edges that would normally occur with sutures. 

### 3.1. “Uneventful” Healing—Photobiomodulation

Of significance to both post-surgical tissue healing, and the sub-ablative application of laser photonic energy, is the phenomenon of photobiomodulation (PBM). Photobiology is the scientific study of the interactions of light (non-ionising radiation) and living organisms [19]. Examples of photobiological processes in living cells include photosynthesis, bioluminescence, and indirectly, circadian rhythms. 

PBM therapy (PBMT) through the application of photonic energy at specific wavelengths works on the principle of inducing a biological response through energy transfer. Such non-ablative photonic energy delivered into tissues modulates biological processes within that tissue, and also within the biological system of which that tissue is a component part. It is a source of some debate, however, which concerns the thermal rise in irradiated cells or tissue, bearing in mind that as a consequence of absorption, photonic energy will impart increased target molecular activity. However, within a correct incident dose, PBM has no appreciable thermal effects in irradiated tissue [20].

At a cellular level, the application of PBM and absorption biomolecules, and, where relevant, chromophores such as cytochrome c oxidase, has been suggested to promote modifications in mitochondrial activity—essentially through a shift in cellular metabolism towards an aerobic glycolytic cycle and an increase in the manufacture and extracellular release of NO^●^. Increased cellular activity arising from an optimisation of ATP production and the associated release of ROS together may promote a combination of effects, including activated transcription factors affecting RNA and DNA synthesis, a process positively impacting on cellular repair and healing. Indirect effects including nitrogen oxide liberation through electron transport chain activity will lead to increased local vessel dilation, and increased oxygen availability and cell permeability. Overall, an increase in mitosis and altered cellular autophagy is observed [21].

Tissue-dependant extracellular effects of PBM may include the selective activation of anti-inflammatory cytokine pathways, resulting in the enhanced resolution of acute and chronic inflammation [22], an optimisation of the consequent production of regenerative products such as collagen and bone [23,24,25,26], improvements in lymphatic drainage, and an increase in the availability of O_2_ to tissues consequent to vasodilatation and the ability to induce analgesia [27]. 

**Figure 7:**
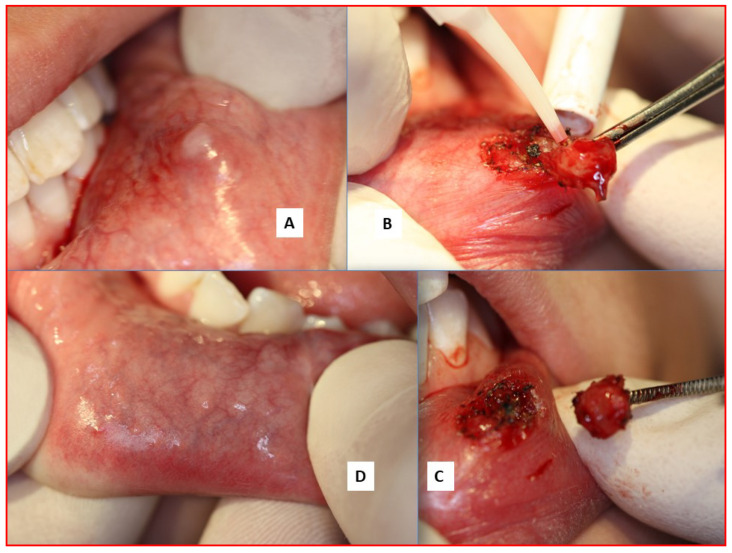
Soft tissue surgery. A mucocele excision, lower lip. The laser used was a diode 980 nm 1.25W CW Fluence 12.2 J/cm^2^. Time taken: two minutes with pauses. A—pre-op, B—excision with haemostasis, C—immediate post-op, D—healing at one month.

Non-surgical pathologies that may respond to PBM therapy include TMJDS, trigeminal neuralgia, oral mucositis, myofacial pain syndrome, herpetic lesions and post-herpetic neuralgia, as well as post-surgery pain management, and also pain associated with various low-grade dental dysaesthesia and that experienced during active orthodontic treatments. Relevant reference sources relevant to the specific dosimetry required, together with the specifics of laser and hand-piece delivery, should be sourced for further detail [28,29,30,31,32,33], although the prescribed dose for many of these conditions in terms of fluence or energy density is between 2–10 Joules/cm^2^, with an appropriate increase in applied “skin dose” to account for deep tissue photon scatter when treating sub-surface structures and conditions [34]. PBM is often cited as the “hidden assistant” during laser soft tissue surgery, in view of photon scatter and power density reductions, and consequent tissue cooling at increasing distances from the surgical site will produce sub-ablative PBM effects. This is considered a significant aspect of the lack of complications following laser soft tissue surgery within the oral cavity. In summary, investigations of the positive effects of PBM have been suggested to suppress inflammatory responses, induce analgesic mechanisms and promote healing [35,36]. Figure 7 demonstrates the haemostatic control, lack of inflammatory response and uneventful healing in laser-mediated minor oral soft tissue surgery.

### 3.2. Laser-Mediated Diagnostics

A number of biological structures have the ability to strongly fluorescence, in which an incident photon of known wavelength is absorbed by the target tissue, then loses a small amount of energy and is re-emitted at a longer wavelength, that may indeed be detectable, either visually or through suitable apparatus [37].

A number of such fluorophores are found in both soft tissue and hard tissue, blood and blood products, and also bacterial plaque and calculus. Accordingly, the application of suitable incident wavelengths may enable the clinician to establish not only the presence, but also the relevance of healthy, unhealthy or unwanted molecular components. This can aid the diagnosis of dental caries, dental calculus and periopathic bacterial plaque, soft tissue health status, and dysplastic or neoplastic changes; additionally, it may give rise to false-negative re-emission in composite restoration material, or alternatively form the basis of photosensitiser-mediated antimicrobial photodynamic therapy. 

A summary of common fluorophores, the incident light wavelength required for them, and the consequent re-emission at longer wavelengths is provided in Table 3.

Additional examples of changes in light frequency, scatter and transmission phenomena may be observed in diagnostic techniques such as doppler flowmetry, diffuse reflectance spectroscopy, optical coherence tomography and Raman spectroscopy [38,39,40,41]. In general, these adjunctive techniques are based upon incident versus re-emission light performance parametry, as applied against known background control healthy human tissue data.

### 3.3. Laser–Tissue Interactions: Dental and Oral Hard Tissues 

The mineral hydroxyapatite is common to dentine and bone, whereas enamel is composed of carbonated hydroxyapatite. Both forms of the mineral are crystalline structures with strong, resistant ionic bonding within the molecule. Dental hard tissue—enamel and dentine—are composite tissues of varying amounts of mineral, protein and water. Dentine, of similar composition to bone, has 45–47% mineral, 20–22% water and 33% protein, whereas enamel has a much higher mineral content (85%), 12% water and 3% protein (mostly found as an inter-prismatic boundary material) [42].

**Figure 8:**
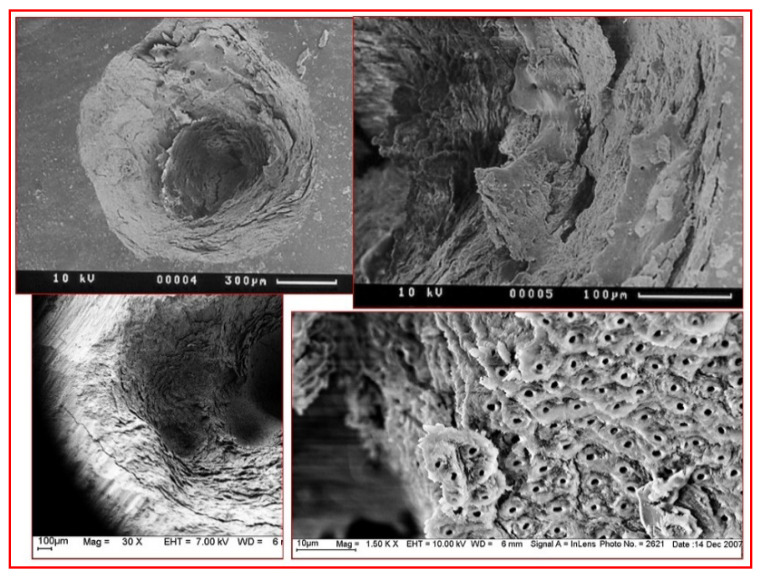
Scanning Electron Microscope (SEM) micrographs of laser-mediated enamel and dentine ablation. Top left and right: the resultant enamel surface is rugged, fragmented and capable of accepting a resin-based composite restoration once any unstable fragments have been removed. Bottom left and right: dentine is rendered smear layer-free, with an intact and stable cut surface.

**Figure 9:**
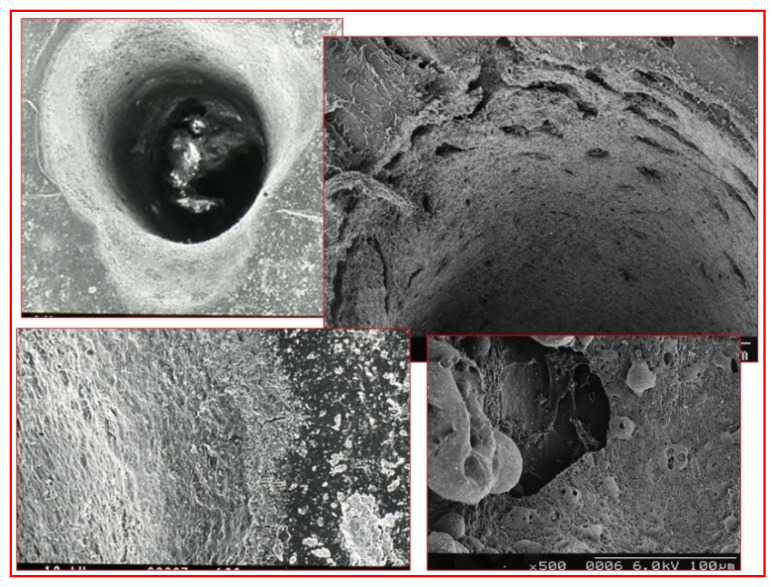
SEM micrograph of laser mediated ablation of bone. Top left, right and bottom left: the use of an erbium laser system enables an accurate and clean ablation without evidence of charring or thermal cracking. Bottom right: this is in contrast to the use of a Nd:YAG system (bottom right), which can result in extensive heat-damage, and melting of the parent hydroxyapatite structure.

Water, either as an interstitial medium or as a source of acidic hydrogen ions (H^+^) and alkaline hydroxide ions (OH^-^), is readily susceptible to vaporisation when applying radiant erbium family wavelengths, and the substantial volumetric change of vaporisation creates a pressure and temperature change that is sufficient to dislocate the crystalline structure and cause a micro-explosive ablation at the point of application—a process known as spallation. The 9300 nm CO_2_ wavelength, although inherently a CW emission, has a tailored micro-gated delivery; hence, there is both some absorption within the water component, but also some targeting of the phosphate and hydrogen phosphate anions of the parent mineral molecule. The surgical management of both dental and osseous tissue requires accuracy and thermal containment in order to avoid unwanted collateral damage. Indeed, the employment of appropriate incident photonic power and laser wavelength with adjunctive water cooling and thermal relaxation will allow for the predictable and selective removal of diseased tissue features. Figure 8 and Figure 9. 

In addition, considerable investigations to address and verify the (often anecdotal) claim of “painless” laser-mediated tooth cavity preparation have been performed. Using visual analogue scoring and randomised clinical trials, a quantification of outcome has helped to define the prospect of anaesthesia-free cavity preparation [43].

**Figure 10:**
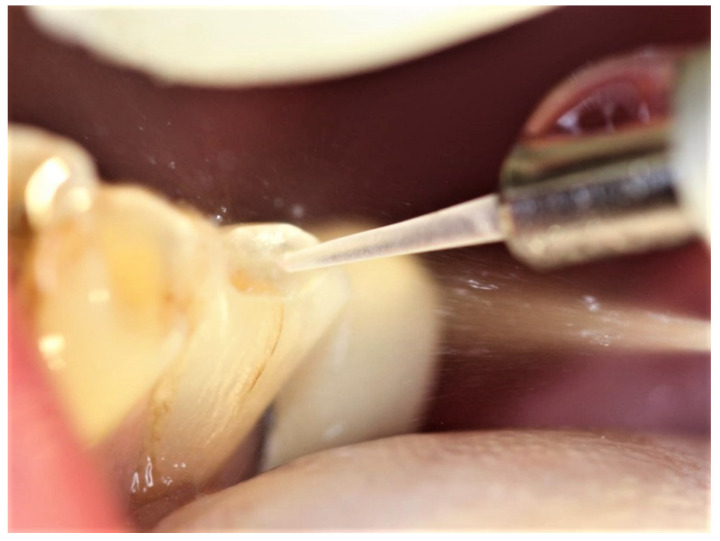
Use of the er YAG laser in tooth cavity preparation. Note the co-axial water spray to aid dispersal of ablation debris and cool the target hard tissue site.

Both erbium and CO_2_ lasers demand co-axial water spray during use in order to disperse the products of ablation, and also to provide adjunctive cooling of the target hard tissue Figure 10. 

## 4. Operating Parametry of Laser–Tissue Interactions

Of practical interest to the clinician in using laser photonic energy, the following factors will each and collectively affect the absorption of laser light by a chosen target tissue [44]:

- Laser wavelength
- Laser emission mode
- Tissue composition
- Tissue thickness
- Surface wetness arising from water or saliva
- Incident angle of the laser beam
- Exposure time.
- Contact versus non-contact modes employed between laser delivery tip and tissue.
- Thermal relaxation factors—Exogenous (water spray, tissue precooling, high-speed suction, pulsing/gating laser emission) and endogenous (tissue type and density, blood supply).

**Figure 11:**
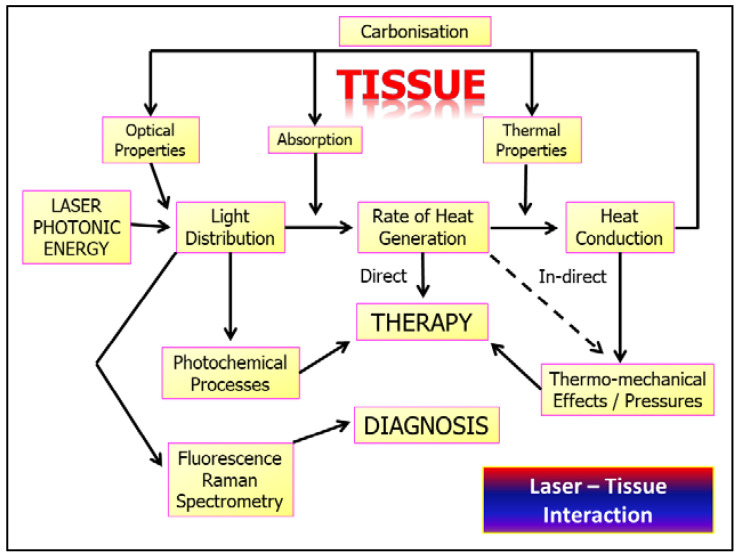
Algorithm depicting the multi-level interaction of varying incident photonic energy as applied to target tissues of quantifiable optical properties. Of prime concern is the thermal containment of laser–tissue interactions. Excessive application of incident power may cause overheating and carbonisation, leading to aberrant interactive effects.

The consequence of such appreciation is to enable the chosen laser therapy to be used, and to avoid the disadvantage of excessive and possibly deleterious thermal increases. Whether the intended use of a laser is diagnostics, sub-ablative PBM or supra-ablative target tissue manipulation, three essential elements are required for careful consideration: (1) the correct or appropriate laser wavelength, and (2) the correct or appropriate light delivery power density and appropriate thermal relaxation process. However, the parameters selected will represent the mainstays of competency on the part of clinicians. Failure to observe such protocols may result in unwanted and damaging collateral thermal rises, but also a change in the optical properties of the target tissue that may indeed alter the optimal laser–tissue interaction desired. This is summarised in Figure 11. 

Concern continues to be expressed over the lack of complete and comprehensive laser operating parameters in published literature to enable the clinician to use laser therapy in order to optimise a desired outcome [45]. 

## 5. Conclusions

Exposing oral and dental tissues to photonic energy has enabled a transformation of how both the assessment of oral health and the modality for treating disease can be effectively achieved. Of key importance to laser–tissue interactions within a predicted therapy “envelope” are demands for the clinician to understand the varying compositions of host tissues, and how these may be managed and manipulated using laser energy without unwanted damaging effects. Laser–tissue interactions may be sometimes inconsistent due to tissue anisotropy and may continue to pose some difficulty for the dental clinician; however, the development of many laser delivery instruments, amounting to a facility to produce laser photonic energy at several wavelengths between the visible and far-IR areas of the electromagnetic spectrum, continue to address many of these inconsistencies.

The current paper outlines the basis of laser–tissue interactions and presents how individual laser wavelengths of varying operating parameters may be applied to interact with target oral and dental tissues.

## Figures and Tables

**Table 1:** Commonly used laser wavelengths associated with dental treatment. Photonic energy and wavelength are inversely related. With ascending numerical values of wavelength, the corresponding photonic energy (expressed as electron volt—eV) is reduced.

(eV)	Laser	λ (nm)
2.8 2.4	InGaN KTP	445 532
2.0	He-Ne	633
1.6	Diode	810
1.2	Nd:YAG	1064
0.4	Er:YAG	2940
0.1	CO_2_	10,600

**Table 2:** Dissociation energy, expressed in eV values, required to break the bonds (covalent, coordinate, etc.) that bind atoms within molecules. Examples represent component molecules within tissue water and biofluids, and also ionic forces within the crystal lattice of hydroxyapatite. Data reproduced with thanks from Mó, O; Yáñez, M, et al. J Phys Chem A. **2005**, 109(19), 4359–4365 [3].

Dissociation Energy of Selected Chemical Bonds	
Type of Bond	Dissociation Energy (eV)
C = O	7.1
C = C	6.4
O-H	4.8
N-H	4.1
C-C	3.6
C-N	3.0
C-S	2.7
Fe-OH	0.35
HA Lattice	310

**Table 3:** Common natural and man-made fluorophores that may be met within clinical dentistry. “Overlapping” excitation—for example, that found in porphyrins as an original component of blood haemoglobin but also a by-product contaminant of dental plaque, calculus and caries, and also tooth discolourations. Source: Original graphics S. Parker. Data reproduced with thanks: Kim, A; Roy, M; Dadani, FN; Wilson, BC; Topographic mapping of subsurface fluorescent structures in tissue using multiwavelength excitation. J Biomed Opt. **2010,** 15(6), 066026 [37].

Fluorophore	Excitation nm	Fluorescence Peak	Comments
Tryptophan	275	350	Amino acid
Collagen	335	390	Connective Tissue (CT)
Elastin	360	410	CT
Keratin	370	505	Surface analysis
Porphyrins	405, 630	590, 625, 635, 705	Cell mitochondria/metallo-, copro-, proto-porphyrins
Healthy Enamel	405	533	
Caries	405, 488, 655	580–700	
Inorganic composites/GI	655	Fluorescence > 700 nm giving rise to false positives	
Calculus/plaque	405, 630	Fluorescence peaks assoc. with porphyrins giving rise to false positives	
